# Social Support and Resilience Among Patients with Burn Injury in Lahore, Pakistan

**DOI:** 10.7759/cureus.867

**Published:** 2016-11-08

**Authors:** Ahmed Waqas, Sadiq Naveed, Mariam M Bhuiyan, Jawad Usman, Ahmed Inam-ul-Haq, Sara S Cheema

**Affiliations:** 1 Final Year MBBS Student, CMH Lahore Medical College and Institute of Dentistry; 2 Psychiatry, KVC Prairie Ridge Hospital; 3 School of Medicine, The Johns Hopkins University School of Medicine; 4 Department of Medicine, FMH College of Medicine and Dentistry, Lahore, Pakistan; 5 Department of Medicine, CMH Lahore Medical College and Institute of Dentistry

**Keywords:** resilience, burns, social support, pakistan, ego resiliency scale, multidimensional scale of perceived social support

## Abstract

**Introduction:**

Social support and ego resiliency play a great role in maintaining the physical and mental health of patients with burn injuries. The present study has been designed to compare ego resiliency levels and the degree of social support in patients with a burn injury and their healthy counterparts.

**Methods:**

This study was conducted in two teaching hospitals in Lahore, Pakistan from May 2015 to July 2015. A total of 80 burn patients presenting in outpatient departments of general surgery, plastic surgery, and burn centers of these hospitals were surveyed conveniently, and for comparison, 80 patients presenting in outpatient departments with minor ailments, for routine checkups or follow-ups were recruited. The questionnaire comprised three sections: demographics, the Urdu versions of the Ego Resiliency Scale (ER-89), and the Multidimensional Scale of Perceived Social Support (MSPSS). All data were analyzed in SPSS v. 20 (IBM Corp., Armonk, NY).

**Results:**

Patients with a burn injury were associated with lower scores on the social support scale as well as its subscales assessing support from the significant other, family and friends than their healthy counterparts. However, no significant differences in scores on the ego resiliency scale were reported between these two groups.

**Conclusion:**

Patients with a burn injury perceived low social support levels from society, which negatively affects their health outcomes. However, their resilience levels were not significantly different from their healthy counterparts.

## Introduction

In Pakistan, incidents of stove burns, domestic abuse, accidental burns, and vitriolage are at a constant rise. Accidents and domestic issues are frequently cited as the main reason for burn injuries in Pakistan [1]. Farooq, et al. have reported a very high incidence of unintentional burns in Rawalpindi, Pakistan, which was associated with mishandling of kerosene pressure stoves, ignorance of safe usage techniques, illiteracy, and poor socioeconomic conditions [2]. Highlighting the issue of intentional burns, the Progressive Women’s Association has documented 7,800 cases of women who were deliberately burnt, scalded or subjected to acid attacks in the Islamabad area [3].

Many studies have reported a very high incidence of mental morbidity in these victims and also identified the risk factors in their genesis but most of these studies were from the developed world [4]. A very high incidence of depression, sleeping disorders, low quality of life, sexual dysfunction, anxiety, substance abuse, post-traumatic stress disorder and agoraphobia have been reported in these patients [5-6]. These incidents, not only scar the physical appearance of an individual but also impact one’s mental well-being, self-esteem, and social support [4].

Social support refers to the acceptance, care, and support from the significant other, family, and society [7-8]. It plays an imperative role in both the physical and mental health of the general population, and research conducted in different populations has repeatedly shown its benefits and relevance to the clinical practice. For instance, Chu, et al. found a positive association between perceived social support and well-being in a sample of children and adolescents [9]. It is not surprising that low levels of social support have been found to be associated with a variety of psychopathologies such as post-traumatic stress disorder and anxiety [10], maternal depression [11] and general well-being [9]. Similarly, Grav, et al. [12] found that the Norwegians reporting low levels of emotional and tangible support were more likely to experience depressive symptoms. Previous research has shown that people with high social support are less likely to suffer from negative health consequences even after stressful events such as chronic heart diseases [13].

Similar to social support, ego resiliency plays a great role in maintaining physical and mental health. It refers to the ability to adapt to constantly varying situations and regulate emotions effectively [14]. The presence of attachment-related anxiety, rumination, and negative affect predicts lower rates of ego-resiliency [14]. Ego-resilient individuals are able to be optimistic and find positive meaning when faced with a problem compared to ego-brittle ones. Ego-resilient individuals are intelligent, resourceful and thus, adaptive in stressful situations.

Previous studies in this field have focused on the concept of social support and ego resiliency as a predictor of psychological morbidity among patients of burn injury. However, there is a paucity of studies comparing social support network and resilience levels among these patients and their healthy counterparts. Therefore, this study has been designed to compare ego resiliency levels and the degree of social support in patients with a burn injury and their healthy counterparts. It is hoped that the results of this study will help health care professionals, social workers, and policy makers understand the mental health needs of burn patients and thus, provide them with a better, inclusive, and holistic care.

## Materials and methods

This study was conducted in two teaching hospitals in Lahore, Pakistan from May 2015 to July 2015. A total of 80 burn patients presenting in outpatient departments of general surgery, plastic surgery, and burn centers were surveyed conveniently, and for comparison, 80 patients presenting in outpatient departments with minor ailments, routine checkups or follow-ups were recruited. This study was approved by the Ethical Review board of CMH Lahore Medical College and Institute of Dentistry, Lahore Cantt, Pakistan. All participants had consented to participate in this study.

The questionnaire comprised three sections: demographics, the Urdu version of the Ego Resilience Scale (ER-89) and the Multidimensional Scale of Perceived Social Support (MSPSS). In order to determine ego resilience, the Ego Resilience Scale (ER-89) developed by Block and Kremen in 1996 [14] was used. Its Urdu translated version has been validated in the Pakistani population [15-16]. It consists of 14 items recording responses on a four-point Likert scale. Mean scores are calculated, with increasing scores corresponding to a higher ego resiliency level. The Urdu version of the MSPSS, validated in a South Asian population was used to assess perceived social support levels among the respondents [17]. The MSPSS is a 12-item scale that uses a seven-point Likert scale format (1 = very strongly disagree; to 7 = very strongly agree). It assesses perceived support from three sources: family, friends, and significant other (four items each). The total scores range from 12 to 84, and higher scores indicate higher social support.

All data were analyzed in SPSS v. 20. Frequencies and descriptive statistics were run for demographic variables. A Chi square test was used to analyze if there were significant differences in the proportion of gender, ethnicity, household income, education, and background. A T-test for independent samples was used to analyze differences in age of controls and burn patients. Then, point biserial correlation was used to analyze association of type of case (dichotomous variable: burn/control) with scores on ER-89, MSPSS scale and its subscales.

## Results

The study included a total of 80 patients with burn injuries and 80 healthy controls. The mean age of all recruited subjects was 34.94 years (11.2). The mean duration of time since burn injury for patients was 6.29 (4.7) years. The mean score standard deviation (SD) on MSPSS was 4.64 (1.1); significant other subscale 4.67 (1.3), family subscale 4.70 (1.2) and friend subscale 4.14 (1.2). The mean score on the ego resiliency-89 scale was 2.82 (.63). There were no significant differences in the proportion of gender, education level, and background between patients with burn injury and their healthy counterparts (Table 1). However, significantly higher number of controls were of Punjabi ethnicity and had low income as compared to patients with burn injuries (Table 1). There was no significant difference in the age of healthy controls and burn cases (t=.91, P=.365).

**Table 1:** Demographic characteristics of burn patients and control cases * denotes P > .05, ** denotes P < .001

		Burn		Control		
Variable		Count	Percentage (%)	Count	Percentage (%)	Chi square value
Gender	Male	24	30.0%	15	18.8%	2.8*
	Female	56	70.0%	65	81.2%	
Household income	Low	36	45.0%	63	78.8%	19.3**
	High	44	55.0%	17	21.2%	
Ethnicity	Punjabi	37	46.2%	66	82.5%	22.9**
	Other	43	53.8%	14	17.5%	
Education	Illiterate or primary level	22	27.5%	21	26.2%	.032*
	Higher than primary level	58	72.5%	59	73.8%	
Background	Rural	14	17.5%	15	18.8%	.042*
	Urban	66	82.5%	65	81.2%	

Our results showed that patients with a burn injury were associated with lower scores on the social support scale as well as the subscales of support from the significant other, family, and friends. However, no significant association was found between the type of case (dichotomous variable, i.e. whether the patients were burn victims or had some minor ailment) and scores on the ego resiliency scale (Table 2).

**Table 2:** Point biserial correlation between type of case (burn/control) with scores on MSPSS and ego resiliency scale * denotes P < .001, ** denotes P > .05

Variable	Significant other	Family	Friends	Social support	Ego resiliency
Type of case	.456*	.478*	.281*	.455*	.147**

## Discussion

Our study revealed that burn patients reported lower scores on social support from their significant other, family and friends than their healthy counterparts. This finding is very concerning given the benefits of social support not just in the general population, but also among burn survivors specifically. Previous literature has identified that support provided by family and peers plays a significant role in youth adjustment after burn injury [18] and it also greatly helps children cope with stressors [19].

Stress has a negative effect on wound healing because it causes decreased levels of interleukin-1 alpha and interleukin-8, which are crucial for a patient’s response to injury [20]. Strong social support influences the burn survivors’ self-esteem and can serve as a buffer against the trauma of the burn and thus moderate the effects of stress and improve wound healing [21]. This buffering effect of social support on stress has also been shown in patients facing other catastrophic events. For example, social support is well-known to buffer against stress and independently reduce emotional distress in cancer patients. Most strikingly, social support has been found to correlate with survival in patients with a massive burn injury [20]. Therefore, the lack of social support among burn victims should be a huge concern because it can negatively influence their survival, physical and mental health.

Not only does social support as a whole improve outcomes in burn patients but each of the components of social support has also been individually shown to play crucial roles in their healing. Patterson, et al. [22] found that being married (or living with a significant other) was associated with better adjustment after a burn injury. Family relationships have been found to positively influence psychological adjustment after burn injuries [7, 18, 23-24] and peer support plays an important role as well [25]. In our study, burn survivors perceived decreased social support from their families, friends and significant others than their healthy counterparts.

Perhaps the gravity of these results is augmented by the fact that there were no significant differences in mean scores on the ego resiliency scale between the burn victims and their healthy counterparts. This is perhaps not surprising because it has been seen that despite experiencing catastrophic events, many people maintain hope and resilience. In a similar context, Somasundaram and Devamani [26] reported that cancer patients manifested remarkable rates of resilience despite their poor prognosis.

According to our analysis, there is a positive association between ego resilience levels and perceived social support. There were no significant differences in resiliency between burn patients and their healthy counterparts. Despite this, burn patients reported low levels of social support from families, friends, and significant others. Therefore, it is very likely that burn patients truly had low levels of social support, rather than due to poor resilience, low self-esteem, and poor coping skills [27].

Thus, the care of burn patients should not only focus on treating their wounds, but also involve families, significant others, and friends in a more holistic approach. Better resources should be designed to educate families, significant others, and friends on the physical and mental health effects of burn injuries. Educating the social support network as well as better psychological care may help improve the overall clinical outcome of burn patients [28]. Previous literature has also identified that forming support groups for burn patients and their loved ones, in order to cultivate friendships with others who have gone through similar experiences may also yield better outcomes [29]. It is hoped that the results of this study as well as further research in this field would help health care professionals, social workers, policy makers, and patient’s loved ones to improve physical and mental health of burn patients.

The results of this study should be interpreted with caution. Participants included in this study were recruited from two teaching hospitals in Lahore, Pakistan, hence limiting the generalizability of these results. Inferences related to temporality and causality are limited by the design of this study. There were no apriori estimates to conduct power analyses and minimum sample size calculations for this study. Therefore, more studies are encouraged to elucidate the importance of resilience and social support among patients with burn injuries.

## Conclusions

Patients with a burn injury perceive low social support levels from society, which negatively affects their health outcomes. However, their resilience levels were not significantly less than their healthy counterparts. Culture sensitive rehabilitation programs should be designed to address mental health concerns of these patients as well as to improve their social support networks and resilience levels.
